# Pharmacokinetic Properties of the Nephrotoxin Orellanine in Rats

**DOI:** 10.3390/toxins10080333

**Published:** 2018-08-17

**Authors:** Deman Najar, Börje Haraldsson, Annika Thorsell, Carina Sihlbom, Jenny Nyström, Kerstin Ebefors

**Affiliations:** 1 Department of Physiology, Institute of Neuroscience and Physiology, Sahlgrenska Academy, University of Gothenburg, 41390 Gothenburg, Sweden; deman.najar@neuro.gu.se (D.N.); borje.haraldsson@gu.se (B.H.); jenny.nystrom@gu.se (J.N.); 2 Proteomics Core Facility, Sahlgrenska Academy, University of Gothenburg, 41390 Gothenburg, Sweden; annika.thorsell@gu.se (A.T.); carina.sihlbom@gu.se (C.S.)

**Keywords:** orellanine, clearance, fungal toxin, half-life

## Abstract

Orellanine is a nephrotoxin found in mushrooms of the Cortinarius family. Accidental intake of this substance may cause renal failure. Orellanine is specific for proximal tubular cells and could, therefore, potentially be used as treatment for metastatic renal cancer, which originates from these cells. However, more information is needed about the distribution and elimination of orellanine from the body to understand its potential use for therapy. In this study, 5 mg/kg orellanine (unlabeled and ^3^H-labeled) was injected intravenously in rats (Wistar and Sprague Dawley). Distribution was measured (Wistar rats, *n* = 10, *n* = 12) using radioluminography and the highest amount of orellanine was found in the kidney cortex and bladder at all time-points investigated. The pharmacokinetic properties of orellanine were investigated using LC-MS/MS and β-scintillation to measure the amount of orellanine in plasma. Three groups of rats were investigated: control rats with intact kidneys (*n* = 10) and two groups with bilateral renal artery ligation (*n* = 7) where animals in one of these groups were treated with peritoneal dialysis (*n* = 8). Using LC-MS/MS, the half-life of orellanine was found to be 109 ± 6 min in the controls. In the groups with ligated renal arteries, orellanine had a half-life of 756 ± 98 min without and 238 ± 28 min with dialysis. Thus, orellanine was almost exclusively eliminated by glomerular filtration as well as by peritoneal dialysis.

## 1. Introduction

Orellanine is a natural toxin found in the *Cortinarius* family of mushrooms found in North America and in Europe. Its selective renal toxicity was recognized already in the 1950s [1]. Each year, several people suffer from renal damage ranging from reduced to complete loss of renal function by accidentally ingesting the mushroom and there is still no specific antidote to orellanine poisoning. It is well known that orellanine specifically targets the tubular epithelium [2,3], but the toxicological properties and mechanisms are still not fully known. Several potential mechanisms have been described and all of them point towards oxidative stress [3,4,5,6]. Data from our group suggest that orellanine nephrotoxicity in vivo is mediated by a combination of increased oxidative radical formation and orellanine-induced down-regulation of several intracellular anti-oxidative enzymes [3]. Since orellanine specifically targets the tubular epithelial cells, our group has suggested that it could be used for treatment of metastasizing renal cancer originating from the tubular epithelium [7,8].

**Figure 1:**
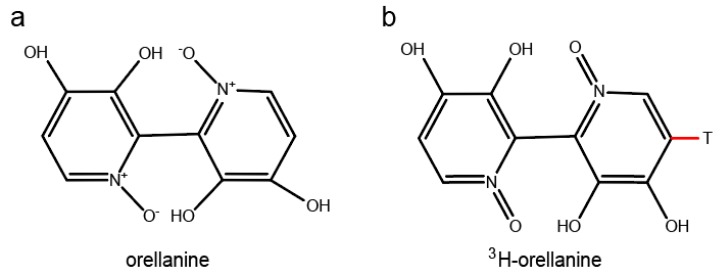
The structural formula of orellanine (**a**) and structure of ^3^H-labeled orellanine (**b**).

Orellanine is a bipyridine *N*-oxide (3,3’,4,4’-tetrahydroxy-2,2′-bipyridine-*N*,*N*’-dioxide) with a molecular weight of 252.19 g/mol. When purified, it is a colorless fine crystalline substance. The structure of orellanine was first described in 1979 [9]. In 1985, the photodecomposition of the compound was described by the same group [10] and it was synthesized in the same year [11]. Orellanine decomposes when heated above 150 °C, when exposed to UV, or by reacting with hydrogen in the presence of platinum as a catalyst. If orellanine is reduced, it yields through the toxic form orellinine, the nontoxic substance orelline with the structure of 3,3’,4,4’-tetrahydroxy-2,2’-bipyridyl and molecular weight of 220.18 g/mol. Orellanine has four pKa values at approximately 0.5, 1.0, 7.0, and 7.4. The net charge at physiological pH is close to −4. The structure of orellanine is shown in Figure 1. In mushrooms, orellanine mainly exists in its di-glucosylated form known as orellanine-4,4’-diglucopyranoside [7,12,13]. Small amounts of orellinine and orelline compared to the amount of orellanine are also detected in the mushrooms [14]. Intoxication with orellanine varies in severity depending on the dose ingested but two to three mushrooms have been estimated as enough to develop dialysis dependent kidney failure [15,16]. Studies of intoxication with orellanine in rats show no signs of acute toxicity apart from renal failure and no sign of damage to organs other than the kidney [17]. In a retrospective case control study, orellanine-intoxicated patients where compared to patients with renal failure due to other causes. No differences between the groups were seen in damage to other organs or in cause of death [18]. It is known that orellanine is excreted from the body into the urine during the first 24 h after intake [17] and that plasma levels are undetectable a few days after intoxication [19]. In contrast, there is a case report of high orellanine concentration (6.12 mg/L) measured 10 days after the suspected intake of mushrooms [20]. No study of the pharmacokinetic properties of the substance has been published as of yet.

The primary aim of this study was to perform a pharmacokinetic and distribution study of orellanine in rats to further explore if the toxin could have a future as a therapeutic option for treating renal cancer. Unlabeled and ^3^H-labeled (tritium) orellanine were intravenously administrated to anesthetized healthy rats with or without renal function (ligated and un-ligated kidneys), including one group without renal function, but on dialysis, and the elimination from plasma was measured. The ^3^H-labeled orellanine was used to capture elimination of orellanine and any metabolites formed in the rats. ^3^H-labeled orellanine was also used for quantitative whole body radioluminography to measure the distribution of orellanine in different organs after administration. The results are relevant for our understanding of the dynamics of orellanine intoxication and for future potential therapeutic clinical use of orellanine in treating patients with metastatic renal cancer.

## 2. Results

### 2.1. Radioluminography

**Figure 2:**
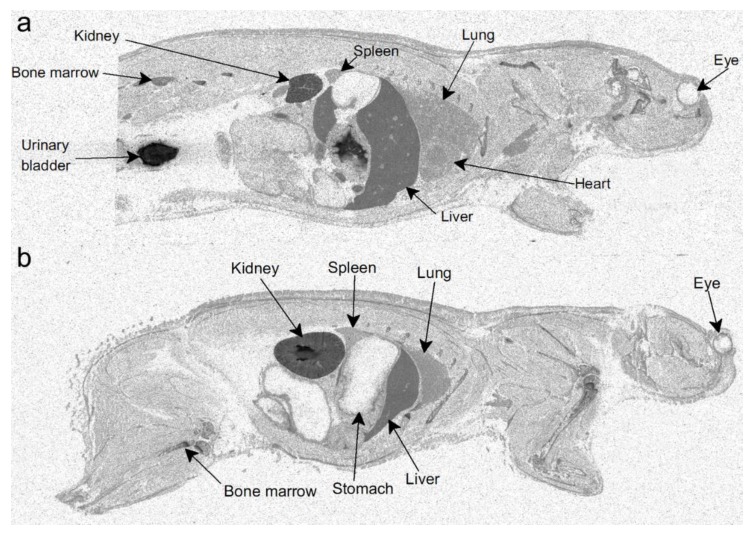
Distribution of orellanine after injection of ^3^H-labeled orellanine in rats shown by radioluminography. Rats injected with a single dose of ^3^H-labeled orellanine 30 min after injection. (**a**) shows the highest radioactivity in the kidney and the bladder. Rats that first received a single dose of orellanine 72 h before injection with ^3^H-labeled orellanine showed the same pattern with the highest radioactivity in the kidney and bladder after 30 min (**b**).

In order to investigate the distribution of orellanine in the body, two setups of radiolumionography experiments were conducted. In the first experiment, rats were injected with a single dose of ^3^H-labeled orellanine (see Figure 1 for structure of orellanine and ^3^H-labeled orellanine). Rats were then sacrificed at 0.5, 1, 6, 12 and 24 h. At all time-points, the highest concentration of orellanine was found in the urinary bladder (at 0.5 h with 560 nmol-eq/g tissue) and the kidney cortex (at 0.5 h with 76 nmol-eq/g tissue, see Table 1). The radioactivity in the blood declined from the highest concentration of 8.6 nmol-eq/g tissue at 30 min after administration to 1.3 nmol-eq/g at the last time point. High concentrations of radioactivity compared to blood was also found at the first time point in the liver (35 nmol-eq/g tissue), the bone marrow (15 nmol-eq/g tissue), and the connective tissues (9.7 nmol-eq/g tissue). Note that the radioactivity signal cannot discriminate between orellanine and any metabolites formed. The other organs investigated had lower radioactivity than the blood at all-time points studied (see Table 1 and Figure 2a). In order to investigate if reduced renal function and repeated exposure to the toxin affected the distribution pattern, a second set of experiments was performed. In this set, the rats were pre-treated with a dose of unlabeled orellanine before administration of the radioactive labeled substance. The results obtained were similar to the single dose experiment with the highest levels of radioactivity seen in the kidney cortex and in the urinary bladder (see Table 2 and Figure 2b).

### 2.2. Pharmacokinetics Study: General Condition of Animals

For the pharmacokinetic study of orellanine in rats, three groups of animals were used. One group of rats has an intact kidney function and two groups had ligated renal arteries to remove kidney function. One of the groups with ligated kidneys underwent dialysis as renal replacement therapy and dialysis was initiated immediately after administration of orellanine. Plasma was collected at 10, 30, 45, 60, 90, 180, and 360 min after administration of orellanine. The body weights of the animals were similar in the three groups, which are 363 ± 54 g, 333 ± 43 g, and 325 ± 61 g for the controls including rats with ligated renal arteries and rats with ligated renal arteries and dialysis respectively (*n* = 10, 7, and 8). The mean arterial blood pressure (MAP) started at similar levels. Thus, MAP was 94 ± 8 mmHg in the control group, 94 ± 4 mmHg in the ligated group, and 92 ± 7 mmHg in the group with ligated kidneys undergoing dialysis. After more than 6 h of anesthesia, the animals had lower MAP values, which were 81 ± 18 mmHg, 79 ± 13 mmHg, and 65 ± 14 mmHg, respectively. For the rats undergoing dialysis, the dialysis resulted in a slight net removal of fluid (ultrafiltration), which likely explains the lower MAP in that group. However, the animals were all in good condition and the MAP levels were acceptable for all animals, which is shown by the highly efficient dialysis (see below).

### 2.3. Parallel Reaction Monitoring (PRM) of Orellanine

Targeted tandem mass spectrometry (LC-MS/MS) analyses were used to determine the profile of orellanine elimination over time in all groups. The linearity for the method was determined and the response of orellanine in the standard curve showed a linearity in the examined concentration range of 0.039 µg/mL to 15 µg/mL.

In the analysis of the study samples, a reference plasma sample was analyzed between the time series from the different animals. The reference sample contained a known amount of orellanine and was used to roughly estimate the orellanine concentrations in study samples and was used to enable a comparison between animals and groups. The lowest level of orellanine was observed in the control group at all time points while higher levels were observed in the animals without renal perfusion and without urine production (data not shown).

**Figure 3:**
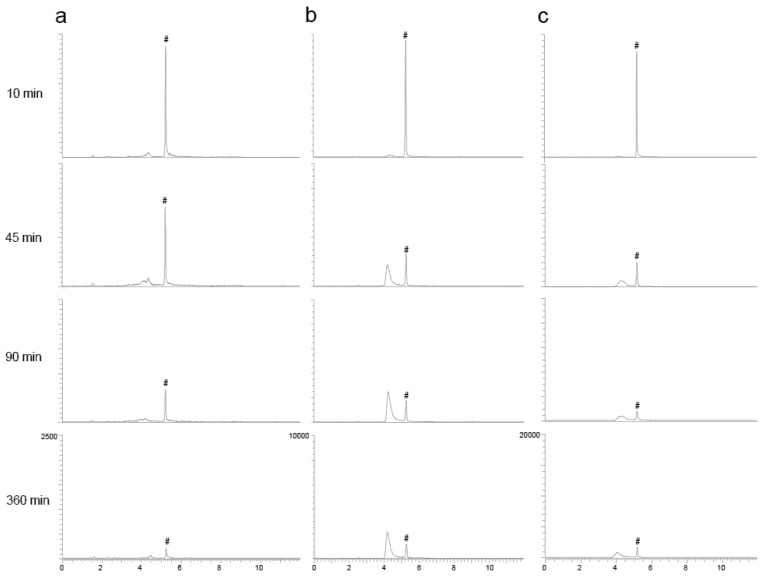
Representative extracted LC-MS/MS chromatograms from the analysis of plasma samples. Orellanine (#) has a retention time at 5.2 min. The orellanine peak is well separated from the metabolite peak seen with retention times around 4 min. Study samples from one representative animal with intact renal function (**a**) ligated kidneys (**b**) and ligated kidneys and dialysis (**c**) at four different time points after iv administration of orellanine. Metabolites are formed with time and rats with ligated kidneys and no urine production form most metabolites.

**Figure 4:**
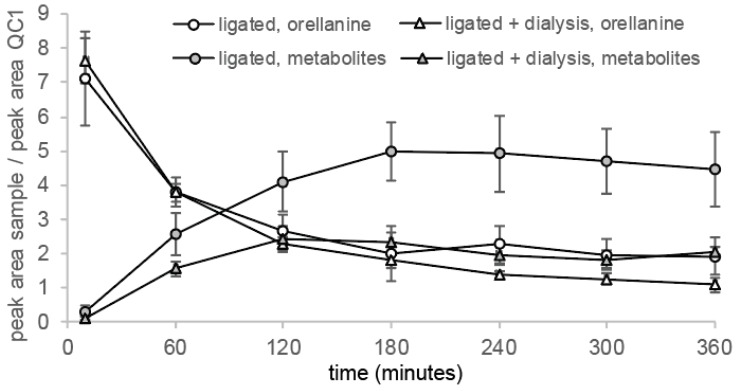
Formation of metabolites correlates with the elimination of orellanine. Rats with ligated kidneys and therefore no urine production with and without undergoing dialysis showed formation of the orellanine metabolites with time. This correlated with the profile of orellanine elimination. Rats that did not undergo dialysis showed higher levels of metabolites than the rats undergoing dialysis.

The peak in the extracted chromatogram corresponding to the orellanine peak in the different groups and in the reference eluted at a retention time (RT) of 5.2 min (Figure 3). Unexpectedly, peaks at a RT around 4 min were also detected in the samples from a later time point. During the PRM analysis, full fragment ion spectrum of orellanine (*m/z* 253.04 Da) was monitored continuously throughout the entire LC separation and the most intense fragment (*m/z* 236.2 Da) was extracted for the quantitation. Inspection of the full fragment spectra corresponding to orellanine and the additional peaks revealed identical fragment ion spectra (data not shown). These findings suggest a time-dependent formation of orellanine metabolites that are in-source fragmented during the analysis and, therefore, detected as orellanine. To confirm that the early eluting peaks are due to ion source fragmentation, parameters in the MS-method were set to minimize ion source fragmentation. As a result, the intensity of these peaks was significantly decreased and it was verified that the metabolites are unstable during the ionization in the analysis. Rats without kidney function (ligated kidneys and ligated + dialysis) had different profiles of metabolites than the control group, which indicates elevated amounts of metabolites remaining in the blood in these groups. Figure 4 shows how the formation of the metabolites in rats correlate with the decrease of orellanine levels over time in the ligated animals.

### 2.4. Plasma Concentration of Orellanine Versus Time and Half-Life

In order to investigate the level of orellanine in the plasma of the animals, two different methods were used: LC-MS/MS and β-scintillation. One difference between the methods is that LC-MS/MS detects orellanine while β-scintillation detects all molecules containing the ^3^H-label and therefore cannot differentiate orellanine from any metabolites or break down products.

**Figure 5:**
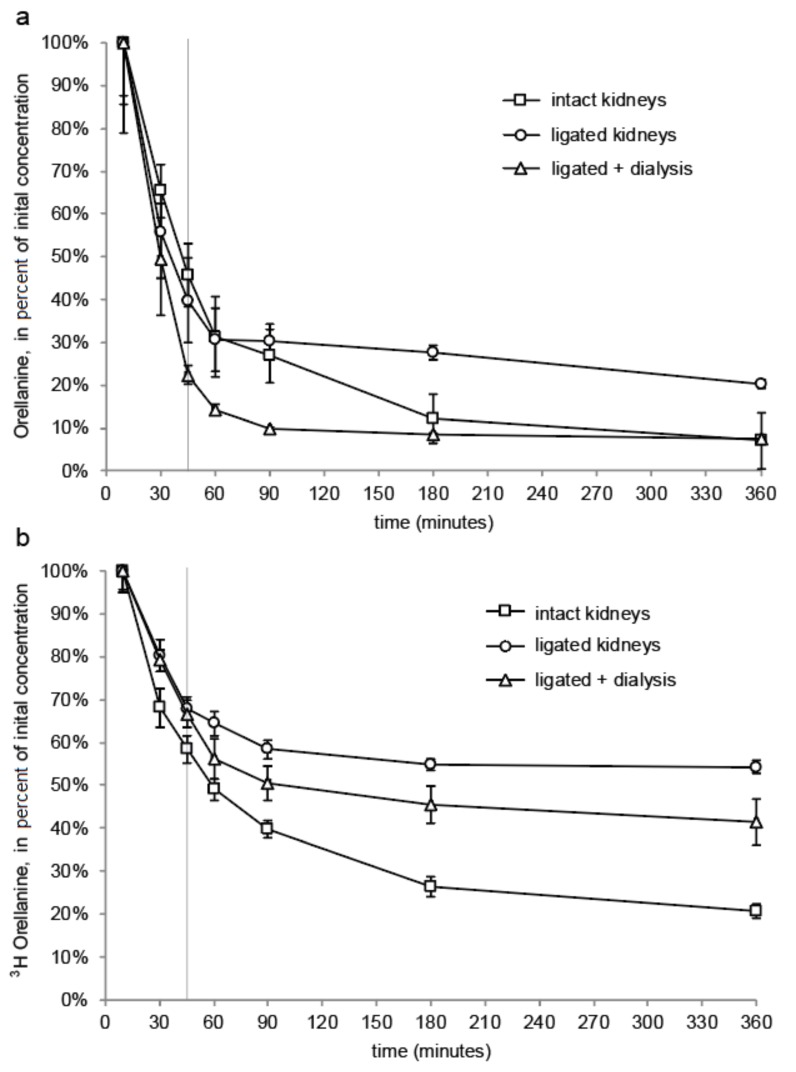
Concentration of orellanine over time presented as a percentage of the concentration measured at 10 min from LC-MS/MS analysis (**a**) and ^3^H-labeled orellanine and its corresponding metabolites by β-scintillation (**b**). The graphs can be divided into two parts with the first part showing the distribution phase and the second part showing the elimination phase. Measurements of orellanine using LC-MS/MS show that rats with intact kidney function have the most efficient elimination under the elimination phase when compared to rats without renal function (ligated kidneys). For rats without renal function, rats not undergoing dialysis have the highest amount of orellanine left after 360 min when compared to rats without renal function but undergoing dialysis. ^3^H-labeled orellanine determined the concentration of both orellanine and its corresponding metabolites using beta scintillation. After 360 min, the rats with intact kidneys have the lowest levels of orellanine and its metabolites left.

Orellanine levels measured by LC-MS/MS corresponds to the elimination of orellanine from plasma. After 60 min, the orellanine levels had decreased more than 50% in all groups due to the orellanine being taken up by renal cells or ending up in the urine, which is shown in the distribution experiments. Dialysis was initiated for one group of rats with ligated kidneys at time point 0 and the first change of dialysis fluid took place at +45 min. This renders a larger distribution volume in these rats and, therefore, the concentration of orellanine in the blood is the lowest in this group at time points 30 min and 45 min. After 360 min, rats with intact renal function and the rats with ligated kidneys receiving dialysis had eliminated most of their orellanine from the blood. The rats with ligated kidneys still had over 20% of the initial concentration left after 360 min (see Figure 5a).

Measurements of ^3^H-labeled orellanine levels in the plasma showed a slower elimination during the distribution phase (0–45 min) than the LC-MS/MS measurements of orellanine. After 360 min and after measurement with beta scintillation of ^3^H-labeled orellanine, all three groups of rats had higher amounts of the initial dose of orellanine left than when measuring orellanine with LC-MS/MS. The difference is due to measurements of the radioactive substance reflecting both orellanine and metabolites being formed. The half-life of orellanine was determined using data obtained from the time points between 45 min to 360 min. The measurements using LC-MS/MS resulted in half times of orellanine being 109 ± 6 min for rats with intact kidneys, 756 ± 98 min for rats with ligated kidneys, and 238 ± 28 min for rats with ligated kidneys undergoing dialysis. Measurements with beta scintillation using ^3^H rendered half times for orellanine and its metabolites of 225 ± 10 min, 1033 ± 183 min and 583 ± 30 min, respectively (see Table 3).

### 2.5. Clearance of Orellanine

The clearance of orellanine was calculated from Equation 3 (see Methods section). The average clearances were 483 ± 24 µL/min for the controls, 75 ± 10 µL/min for rats with ligated renal arteries, and 251 ± 47 µL/min for ligated animals on dialysis. The differences between the groups are all statistically significant (*p* < 0.05).

The protein binding of orellanine was determined for plasma from different species and found to be 33.5% for rats (unpublished data). Correcting for this degree of protein binding, the clearance for ´free, unbound´ orellanine was calculated. The renal clearance of orellanine was estimated as the difference between the clearance of the controls and the rats without renal function (i.e., including the effect of ligating renal arteries and protein binding), which resulted in an average value of 613 ± 52 µL/min. Similarly, the clearance under acute peritoneal dialysis was determined as the difference between the rats with ligated renal arteries with and without dialysis and was 263 ± 86 µL/min.

The clearance of orellanine and its metabolites was determined from the elimination of ^3^H-labeled orellanine using similar calculations as the calculations of orellanine in the LC-MS/MS experiments. The average clearance was 232 ± 9 µL/min for the controls, 63 ± 14 µL/min for rats with ligated kidneys, and 90 ± 5 µL/min for ligated kidneys on dialysis.

Note that the clearance of ^3^H-orellanine in rats with normal renal function was much lower than for orellanine-treated rats (*p* < 0.001) but higher than for ^3^H-orellanine of rats with ligated kidneys without dialysis and rats with ligated kidneys on dialysis (*p* < 0.05), which suggests a higher degree of protein binding for the metabolites.

## 3. Discussion

Although research so far has explored the effects of orellanine toxicity and effects on kidney function [13,21], there is a need for a better understanding of the pharmacokinetic properties of this nephrotoxin. This might help in understanding future deadly webcap poisoning cases in improving clinical management [18] as well as opening the way for orellanine as a potential cure for metastatic end stage renal carcinoma [8]. New and curative therapy options for this type of cancer is needed since the outcome for patients with metastatic disease still is poor even though several therapeutic options have been suggested [22,23,24,25]. We have shown in an earlier paper that orellanine toxicity extends to the renal carcinoma cells in vitro and to human renal cell carcinomas tumors on rats [8], which indicates that orellanine could have a future as an anti-renal cancer treatment. 

In this study, we have determined the pharmacokinetic properties of orellanine and its metabolites. Our work shows that orellanine is eliminated rapidly from plasma with a half-life of 109 min in anesthetized rats and mainly ends up in the kidney cortex and urine. Reduced renal function in the rats obtained by repeated dosing of orellanine did not affect the distribution pattern. The half-life of orellanine and its metabolites was twice as long (222 min), which most likely reflects a higher degree of protein binding of the metabolites. The renal clearance of orellanine not bound to protein was 613 µL/min, which is roughly 50% of the glomerular filtration rate (GFR) of awake rats reported to be 1310 µL/min [26]. Orellanine was easily removed by acute peritoneal dialysis with a clearance of 263 µL/min. Metabolites of orellanine were eliminated by peritoneal dialysis and by the kidneys but removal is far slower than orellanine.

There are three potential explanations for the clearance of orellanine being lower than the expected GFR level: First, the discrepancy suggests that orellanine is freely filtered across the glomerular barrier to urine where the compound is reabsorbed by proximal tubular cells and returned to plasma. Thus, with a 95% interval of confidence of renal clearance for orellanine of 491–735 µL/min, between 37% to 56% of the filtered orellanine is likely to have been reabsorbed. Second, anesthesia and abdominal surgery may reduce GFR even though the effect is expected to be small with isoflurane as anesthetic and minimal surgical procedures [27,28]. Third, orellanine may acutely reduce GFR by 50% due to a direct toxic effect on nephrons [3,5,29]. However, there are no reports of direct effects of orellanine on the renal vasculature, the glomerular capillaries, or the mesangial cells that could explain such an immediate reduction of GFR. Therefore, the first alternative seems most plausible albeit speculative since independent measurements of GFR are lacking.

The LC-MS/MS analysis suggests a time-dependent formation of orellanine metabolites eluting at a shorter RT compared to orellanine. Both the fragmentation pattern of the metabolites and evidence that their detection is effected by in-source fragmentation suggests that the metabolites are orellanine conjugated with a charged group at the hydroxyl group/s that falls off during the ionization. We were not able to determine the *m*/*z* of the intact metabolites due to the lower ionization efficiency obtained when changing the MS-parameters to also reduce the in-score fragmentation. In a previous study of mushrooms extracts, mono-glucopyranoside and diglucopyranoside were demonstrated to be naturally occurring glucosides of orellanine [7]. Furthermore, these mushroom glucosides were eluting before orellanine as well as had a similar fragmentation pattern as orellanine compared to the metabolites detected in the present study. Hydroxyl groups in aromatic systems are easily conjugated with glucuronic acid, which results in a more polar metabolite compared to the parent compound. These conjugates can be unstable in the ion source and are, therefore, detected as the parent compound in the MS-analysis. Moreover, the glucosides in the mushroom extract were shown to hydrolyze to orellanine in an acidic environment [7]. Re-analysis of the plasma samples after storage in an acidic environment also indicated that these metabolites are hydrolyzed over time. Therefore, we speculate that the metabolites formed in the present study could be glucosides of orellanine formed in the circulation. The two most well-known metabolites of orellanine are orellinine and orelline, but they are not the metabolites found in this experiment.

The slower elimination of orellanine measured with β-scintillation was most likely due to the formation of the metabolites during the experiment, which is shown in Figure 4. In the LC-MS/MS analysis, the elimination of orellanine was monitored while, in the β-scintillation measurement, orellanine and its metabolites cannot be differentiated and both are measured together.

The distribution of orellanine in the rats after injection with the toxin supports a rapid elimination from the blood with most orellanine ending up in the kidney cortex and urine. Other organs displayed higher levels of ^3^H than the blood after 24 h except for the kidney cortex and urine, the liver, spleen, and bone marrow even though these levels were much lower than for the kidney and urinary system. There are no reports of patients with orellanine intoxication having any damage to any other organs except the kidneys [18].

These results seem to suggest the presence of specific renal transporters responsible for uptake of orellanine from urine into tubular cells and possibly back in intact form to blood. Hereby, the half-life may be longer than expected due to a small solute being freely filtered across the glomerular wall. It was not within the scope of this paper to identify the proteins responsible for such uptake of orellanine. However, revealing these transporters will be key for the understanding of the toxicological and potentially therapeutic effects of orellanine.

## 4. Conclusions

In conclusion, orellanine is mainly eliminated by renal excretion involving free glomerular filtration and tubular reabsorption. The compound also forms metabolites and they appear to have stronger protein binding properties when compared to the intact orellanine. Therefore, they remained longer in the system. The metabolites formed are likely to be glucosides of orellanine, which are naturally occurring in orellanine-containing mushrooms. In light of this, we conclude that this nephrotoxic compound may be eliminated rapidly through the urine or by dialysis.

## 5. Materials and Methods 

### 5.1. Test Solution of Orellanine

Pure orellanine was synthesized by Ramidus AB, IDEON Lund, Sweden, as a 99% pure freeze-dried powder without detectable contaminations of its metabolites. The substance was kept dry and protected from light at room temperature. The orellanine was dissolved in 3 M HCl (Sigma-Aldrich, Steinheim, Germany) and the pH was carefully raised by adding portions of small amounts of 10 M NaOH (Sigma-Aldrich, Steinheim, Germany). In this process, the orellanine precipitates before becoming a clear solution. After a clear solution was obtained, the pH was normalized with 3 M HCl to a pH of 7.4 to 7.5. The solution was further diluted in PBS without magnesium and calcium (Lonza, Verviers, Belgium) to obtain an orellanine stock solution of 7.6 mg/mL containing 1 M NaCl. The solution was sterile filtered, aliquoted, and stored in −80 °C until use. The whole process took place in a dark room.

Radiolabeling of orellanine with tritium (^3^H) was done by the Red Glead Discovery AB, Lund, Sweden. The procedure resulted in > 95% bound ^3^H-labeled orellanine with a specific activity of 35.5 Ci per mmol orellanine. The structure of ^3^H-labeled orellanine is shown in Figure 1b.

### 5.2. Rat Experiments

For the radioluminography experiments, male Wistar rats (Taconic, Ebjy, Denmark) were used which weighed, on average, 200 g on arrival. For the pharmacokinetic experiments, male Sprague Dawley rats (Charles River, Wilmington, MA, USA) with, on average, 200 g body weight on arrival were used.

### 5.3. Radioluminography

Two setups of radioluminography experiments were conducted. One setup with one single dose of orellanine (5 mg/kg) was conducted and one setup with two doses was conducted. For the single dose experiment, 10 male Wistar rats were administered ^3^H-labeled orellanine formulated to 0.4 mCi/mL and 1.25 mg/mL in physiological saline intravenously in the tail vein. Two rats were sacrificed at each 0.5 h, 1 h, 6 h, 12 h, and 24 h after administration and one animal per time point was embedded in a gel of aqueous carboxymethyl cellulose and frozen in ethanol at −70 °C. For the second set up with 2 doses of orellanine, 12 male Wistar rats were pre-treated with unlabeled orellanine (1.25 mg/mL), intraperitoneally. After 72 h, the animals were administered ^3^H-labeled orellanine (0.471 mCi/mL, 1.28 mg/mL in physiological saline) intravenously in the tail vein. Three rats were sacrificed at each 0.5 h, 1 h, 6 h, and 12 h after administration of the ^3^H-labeled formulation. For both setups, 30 μm sections were cut at different levels from each embedded animal. The obtained sections were freeze-dried at −20 °C sections and put on ^3^H-imaging plates. Together with the ^3^H calibration standards (^3^H-radioactivity mixed with whole blood), the images were exposed 70 h to 96 h. After exposure on imaging plates, the plates were scanned at a pixel size of 50 μm using BAS 2500 (Fuji Film Sverige AB, Stockholm, Sweden) and quantified using AIDA, version 4.19 (Raytest, Straubenhardt, Germany). For each time point, the radioactivity was determined as the mean value of measurements of three separate sections for each tissue.

### 5.4. Pharmacokinetic Studies

Sprague Dawley rats were randomly divided into three groups including sham operated control rats (*n* = 10), rats with bilaterally ligated renal arteries and hence no urine production (*n* = 7), and rats with ligated renal arteries treated with peritoneal dialysis (PD) (*n* = 8). Rats undergoing PD got a PD-catheter (PE-50, Solomon Scientific, Skokie, IL, USA) inserted into the abdominal cavity. After stabilization of the rats, 5 mg/kg body weight of orellanine containing trace amounts of ^3^H-orellanine was injected into the jugular vein. After a flush of 1 mL glucose-bicarbonate-NaCl solution, a continuous slow infusion of the same solution was started (infusion rate of 17 μL/min, i.e., 1 mL/h). Approximately 400 µL of blood was drawn at the time points: 10, 30, 45, 60, 90, 180, and 360 min. For the group of rats undergoing PD, dialysis was immediately initiated by filling the abdominal cavity with 15 mL of 1.5% glucose solution (Gambro AB, Lund, Sweden). Every 45 min (± 10 min) the PD-fluid was drained and collected. After the experiment, the animals were euthanized with an anesthetic overdose of isoflurane and cardiac excision.

### 5.5. Beta Scintillation of ^3^H

To each of the beta scintillation tubes containing plasma, 3 mL of quenching solution was added. Radioactivity was measured using a Beta Scintillator (Liquid Beckman LS 6500 Scintillation Counter, *Beckman* Coulter Inc., Brea, CA, USA) and the appropriate protocol for tritiated material (^3^H) was used according to the manufacturer.

### 5.6. Bioanalysis of Orellanine 

Plasma samples were filtered through a molecular weight cut-off filter (Nanosep 30k Omega filters, Pall Life Sciences, Port Washington, NY, USA) in order to remove plasma proteins and higher molecular weight biomolecules. Orellanine was extracted from the plasma (20 μL) with the addition of formic acid (final concentration 4% (*v/v*), final volume 84 μL), mixed for 10 min followed by centrifugation (30 min at 1200 rpm). Flow-through samples were collected and transferred into vials. A standard curve within the concentration range 0.039 µg/mL to 15 µg/mL (0.039, 0.78, 0.16, 0.31, 0.63, 1.25, 2.5, 5, 10, and 15 µg/mL was prepared with the addition of orellanine to human plasma. Reference plasma (quality control) samples were prepared with the addition of orellanine to human plasma and final concentrations of 1.25 µg/mL and 5 µg/mL. The linearity was determined to be 0.99 (*r*^2^) within this concentration range. Reference plasma samples and standard curves were filtered in parallel, which is described in Section 5.5. The precision between multiple injections was less than 10% deviation and the accuracy was high with less than 5% between the theoretical and experimental amounts for the reference sample. In the analysis of the study samples, a reference plasma sample was analyzed between the time series from the different animals in each LC-MS/MS run. The reference sample was used to estimate the orellanine concentrations in study samples as well as enable a comparison between animals, groups, and LC-MS/MS experiments. The plasma protein binding of orellanine was determined after 4 h of dialysis (data not shown and found to be quite low, i.e., 33.5% for rat and 42.5% for humans).

The method for LC-MS/MS used was modified from Herrmann et al. [7]. The samples were analyzed using parallel reaction monitoring (PRM) on an LTQ Orbitrap Velos mass spectrometer interfaced to an UltiMate 3000 system (Thermo Fisher Scientific, Waltham, MA, USA). Samples (7.5 µL injection volume) were separated on an Acquity UPLC Peptide CSH C18 column (100 × 2.1 mm, 1.7 µm, Waters, Milford, MA, USA) using a gradient starting with 2.5 min isocratic separation with 5% B followed by a rise from 5% to 60% of B within 1 min and finally made isocratic with 60% B for 2.5 min at 45 °C with a flow rate of 200 µL/min. Mobile phase A was 0.2% formic acid in 2 mM ammonium formate and mobile phase B was acetonitrile in 0.2% formic acid. Orellanine parent ion mass (*m/z* 253.05 Da) was isolated in the ion trap with a 2 Da isolation window. The collision energy was set to 25 with a scan range *m/z* 200.00-260.00. The most intense fragment (*m/z* 236.2 Da, corresponding to loss of 17 Da and OH) was selected for quantification. The peak areas were determined by the extraction and integration of this fragment in the fragmentation spectra using XCalibur (Thermo Fisher Scientific, Waltham, MA, USA) and were used for the determination of the profiles for half-life calculation. Each study sample was injected twice and the average peak area was calculated. The reference samples at 1.25 µg/mL and 5 µg/mL were analyzed before and after each time point from an animal to compensate for day-to-day variation in the analyses and to be able to compare between UPLC-MS runs. The average peak area of orellanine in the study samples was divided by the average peak area for the reference sample at 1.25 µg/mL in the analysis sequence. To roughly estimate the concentrations in study samples, the ratios were multiplied by 1.25 µg/mL.

### 5.7. Pharmacokinetic Analysis

For the pharmacokinetic analysis, a first order kinetic model was used. According to the model, the elimination of a solute after an i.v. injection is determined by an elimination rate constant, k_el_. Thus, the concentration at time t can be estimated using the following expression where C_0_ is the concentration at the time of injection.

(1) $$\;C\left(t\right)\;=\;C_{0}\times e^{-k_{el}\times t}\;$$

The constant k_el_ is dependent on the clearance (K) and the distribution volume (V) and it has been shown that k_el_ = K/V. Rearranging Equation 1 and inserting K/V gives the relationship below.

(2) $$\;\frac{Kt}{V}=\;Ln\left(\frac{C\left(t\right)}{C_{0}}\right)\;$$

Clearance of orellanine can be determined with the equation below.

K = k_el_ × V (3)

For clearance calculations, V was assumed to equal the extracellular fluid volume (ECV) estimated independently of the kinetic modeling from data in the literature [26].

The exact dose of ^3^H-labeled orellanine given was estimated by determining the activity in the injected solution (cpm/mg) and the exact weight of the solution injected. The latter was determined by weighing the syringe before and after injection with 0.1 mg precision. Similarly, the amount of intact orellanine was determined from the concentration of the test solution and the precise injected volume. The half-time is given by Equation (4).

(4) $$\;t_{\text{½}}=\;ln2/k_{el}\;$$

The area under the curve is calculated by the equation below.

(5) $$\;AUC=\int_{t=0}^{t=\infty }Cp\left(t\right)dt$$

The volume of distribution can be derived from Equation (6).

(6) $$V_{D}=\frac{Dose}{AUC\cdot k_{el}}$$

The accuracy of Equation (6) depends on how many h of sampling is done and becomes most accurate for sampling periods of 24 h, which was not technically feasible.

### 5.8. Statistics

Results are presented as the mean ± standard error of the mean. Differences between groups were determined using parametrical tests such as the Student t-test. Statistical significance was defined as *p* < 0.05.

### 5.9. Ethical Approval

All applicable national and institutional guidelines for the care and use of animals were followed. All procedures performed in studies involving animals were in accordance with the ethical standards of the institution at which the studies were conducted. The Regional Ethical Board in Gothenburg, Sweden approved the experiments on 12 June 2012, no. 144–12.

## Figures and Tables

**Table 1:** Individual tissue concentrations (nmol-eq/g tissue) after a single dose of ^3^H-labeled orellanine.

Time Point (h)	0.5	1	6	12	24
Adrenal gland	5.2	2.6	1.1	1.6	<LOQ
Bone marrow	15.0	4.7	3.9	4.0	3.0
Brain	<LOQ	<LOQ	<LOQ	<LOQ	<LOQ
Brown fat	<LOQ	<LOQ	<LOQ	<LOQ	<LOQ
Connective Tissue	9.7	3.2	1.6	2.0	<LOQ
Dermis	3.2	1.4	<LOQ	<LOQ	<LOQ
Epidermis	4.0	1.9	<LOQ	<LOQ	<LOQ
Gastric mucosa	3.2	1.8	1.1	<LOQ	<LOQ
Heart blood	8.6	4.9	2.0	1.7	1.3
Intestinal mucosa	5.3	4.2	0.8	0.8	1.0
Kidney cortex	76.0	73.0	19.0	18.0	15.0
Lens (eye)	<LOQ	<LOQ	<LOQ	<LOQ	<LOQ
Liver	35.0	25.0	7.8	6.1	4.0
Lung	8.0	4.0	1.6	1.4	0.9
Lymph node	3.9	2.1	0.8	14.0	<LOQ
Myocardium	6.0	2.6	1.1	0.8	0.6
Pancreas	3.9	1.9	1.6	1.0	<LOQ
Salivary gland	4.5	2.6	1.2	1.0	0.7
Skeletal muscle	1.8	1.6	<LOQ	<LOQ	<LOQ
Spleen	6.5	5.6	4.3	3.8	3.3
Testicle	1.9	1.3	<LOQ	<LOQ	<LOQ
Thymus	4.9	2.5	1.2	1.1	1.1
Thyroid gland	5.2	2.5	1.7	0.9	0.6
Urinary bladder	560.0	2600.0	200.0	37.0	18.0
Limit of Quantification (LOQ)	0.6	0.6	0.7	0.52–0.67	0.6

Measurements are the mean value of three measurements for each tissue. The CV is 7.5–14.3%.

**Table 2:** Individual tissue concentrations (nmol-eq/g tissue) after a single dose of ^3^H-labeled orellanine 72 h after a single dose of unlabeled orellanine.

Time Point (h)	0.5	1	6	12
Adrenal gland	5.1	8.0	2.1	0.7
Bone marrow	10.0	5.8	6.1	4.4
Brain	<LOQ	<LOQ	<LOQ	<LOQ
Brown fat	<LOQ	1.3	<LOQ	<LOQ
Connective tissue (skin)	10.0	12.0	1.4	0.8
Dermis	2.0	2.0	<LOQ	<LOQ
Epidermis	4.5	6.9	1.9	<LOQ
Gastric mucosa	3.8	4.8	1.9	1.0
Heart blood	9.0	12.0	3.5	1.8
Intestinal mucosa	3.3	9.2	3.2	2.1
Kidney cortex	63.0	90.0	23.0	22.0
Lens (eye)	<LOQ	<LOQ	<LOQ	<LOQ
Liver	29.0	37.0	18.0	7.2
Lung	8.8	10.5	3.0	1.3
Lymph node	2.7	7.0	3.7	2.6
Myocardium	4.7	6.3	2.6	1.8
Pancreas	2.9	5.1	2.5	1.7
Pituitary	3.1	5.6	2.2	2.1
Retina	8.3	11.0	2.4	1.0
Salivary gland	3.8	6.2	3.3	2.3
Skeletal muscle	1.9	2.1	2.0	0.9
Spleen	4.6	8.8	6.8	3.5
Testicle	2.4	2.7	1.0	<LOQ
Thymus	4.0	6.2	4.3	2.2
Thyroid gland	4.5	6.6	2.4	1.2
Urinary bladder	590.0	600.0	93.0	15.0
LOQ	0.6	0.6	0.43–0.65	0.54–0.70

Measurements are a mean value of three measurements for each tissue. The CV is 8.5–10.7%.

**Table 3:** Pharmacokinetics of orellanine calculated from time-point 45 min to 360 min.

Parameter	LC-MS/MS, Orellanine			Beta Scintillation, ^3^H-Orellanine		
	Intact Renal Function	Ligated Kidneys	Ligated Kidney Dialysis	Intact Renal Function	Ligated Kidneys	Ligated Kidney Dialysis
Intercept (nmol/L)	2115 ± 342	3028 ± 610	2521 ± 149	1471 ± 71	2538 ± 115	2773 ± 135
Rate of elimination (nmol/mL/min)	0.00651 ± 0.00032	0.000101 ± 0.00013	0.00338 ± 0.00063	0.0031 ± 0.00012	0.00085 ± 0.00019	0.00122 ± 0.00006
Half-life (t^½^, min)	109 ± 60	756 ± 98	238 ± 28	225 ± 10	1033 ± 183	583 ± 30
Dose injected (nmol)	87.3 ± 1.5	85.4 ± 1.3	86.9 ± 1.2	87.6 ± 1.2	84.9 ± 1.3	87.2 ± 1.1
Area under the curve (AUC, nmol × min/L)	503,870 ± 70,670	3,494,674 ± 783,934	645,606 ± 99,420	821,601 ± 52,808	3,223,824 ± 230,783	2,823,938 ± 123,739
Volume of Distribution (V_D_, mL)	31.7 ± 4.8	35.1 ± 8.6	49.1 ± 4.7	35.3 ± 1.7	37.7 ± 4.9	26.3 ± 1.7
